# When common cognitive biases impact debriefing conversations

**DOI:** 10.1186/s41077-024-00324-0

**Published:** 2024-12-18

**Authors:** Michael J. Meguerdichian, Dana George Trottier, Kimberly Campbell-Taylor, Suzanne Bentley, Kellie Bryant, Michaela Kolbe, Vincent Grant, Adam Cheng

**Affiliations:** 1 Institute for Simulation and Advanced Learning, 1400 Pelham Parkway S, Bronx, NY 10461 USA; 2 Department of Emergency Medicine, NYC Health+Hospitals: Harlem Hospital Center, 506 Malcolm X Blvd, New York, NY USA; 3 NYC Health+Hospitals, 50 Waters Street, New York, NY 10004 USA; 4 Icahn School of Medicine at Mt. Sinai, Gustave L. Levy Pl, Elmhurst Hospital Center, 79-01 Broadway, Queens, New York, NY 10029 USA; 5 National League of Nursing, 2600 Virginia Ave NW, Washington D.C, 20037 USA; 6 Simulation Centre, University Hospital Zurich, Zurich, Switzerland; 7 eSim Provincial Simulation Program for Alberta Health Services, Alberta, Canada; 8 Department of Pediatrics and Emergency Medicine, University of Calgary, 28 Oki Drive NW, Calgary, Canada

## Abstract

Healthcare debriefing is a cognitively demanding conversation after a simulation or clinical experience that promotes reflection, underpinned by psychological safety and attention to learner needs. The process of debriefing requires mental processing that engages both “fast” or unconscious thinking and “slow” intentional thinking to be able to navigate the conversation. “Fast” thinking has the potential to surface cognitive biases that impact reflection and may negatively influence debriefer behaviors, debriefing strategies, and debriefing foundations. As a result, negative cognitive biases risk undermining learning outcomes from debriefing conversations. As the use of healthcare simulation is expanding, the need for faculty development specific to the roles bias plays is imperative. In this article, we hope to build awareness about common cognitive biases that may present in debriefing conversations so debriefers have the chance to begin the hard work of identifying and attending to their potential detrimental impacts.

## Background

In healthcare simulation, debriefing has been heralded as the learning conversation, considered the most essential part of the experience. Effective debriefings are intended to drive new learning outcomes and change behaviors [1, 2]. The execution of a simulation debriefing is cognitively demanding and requires the culmination of multiple sub-tasks [3, 4]. As the debriefer approaches each task, the debriefer’s working memory—defined as a short-term memory that processes comprehension and problem-solving—engages to analyze, interpret, prioritize, and remember the event as well as to focus and organize questions, engage learners, maximize learning outcomes, and facilitate the flow of the conversation [4, 5].

While engaged in the task, the debriefer unconsciously processes the new information from both the experience and the debriefing conversation. That information is blended with long-term internal representations of previous similar experiences to create decisions and judgments [6, 7]. In the ideal circumstance, with sufficient cognitive bandwidth, the debriefer will engage more often in “slow thinking”; a concentrated, intentional process to analyze and interpret the experience and manage the debriefing with the learning group. If the debriefing is a particularly complex experience or engages familiar elements from the debriefer’s past experience, the working memory risks shifting into “fast thinking”. In this instance, the brain employs rapid, effortless, and automated processing that taps into pattern recognition and other cognitive biases to navigate parts of the analysis and interpretation [6, 8]. This can be problematic because cognitive biases may lead to changes in behavior or delivery of content that might negatively impact the learning experience.

Cognitive biases are “decisional shortcuts” that are based on previous experiences and established heuristics held as truths that live within our fast, automated processing. [9] When tapped, these cognitive biases exist within our subconscious and can produce both accurate and inaccurate interpretations of information when making judgments or decisions. When acted on, these biases may result in unintentional favoring or antagonism against a person, idea, group, or thing in a way that is not justified despite best intentions [10]. Multiple cognitive biases engage in our processing and perception of the world and can unconsciously land in debriefing conversations. Cognitive biases act as an umbrella term and include more commonly discussed implicit biases among other biases. Existing literature has not detailed the existence and potential impacts of cognitive biases in debriefing conversations.

The goal of this article is to discuss how cognitive biases can influence debriefer behaviors, debriefing strategies, and debriefing foundations potentially disrupting the learning outcomes of the conversation. As there are hundreds of biases, we aim to identify and describe specific biases that are prone to show up in debriefing conversations and illustrate these influences with examples. By building awareness around how these common cognitive biases manifest, healthcare debriefers can begin recognizing their own biases and start attending to their potentially negative impacts.

### Where cognitive biases arrive in debriefing conversations

**Fig. 1:**
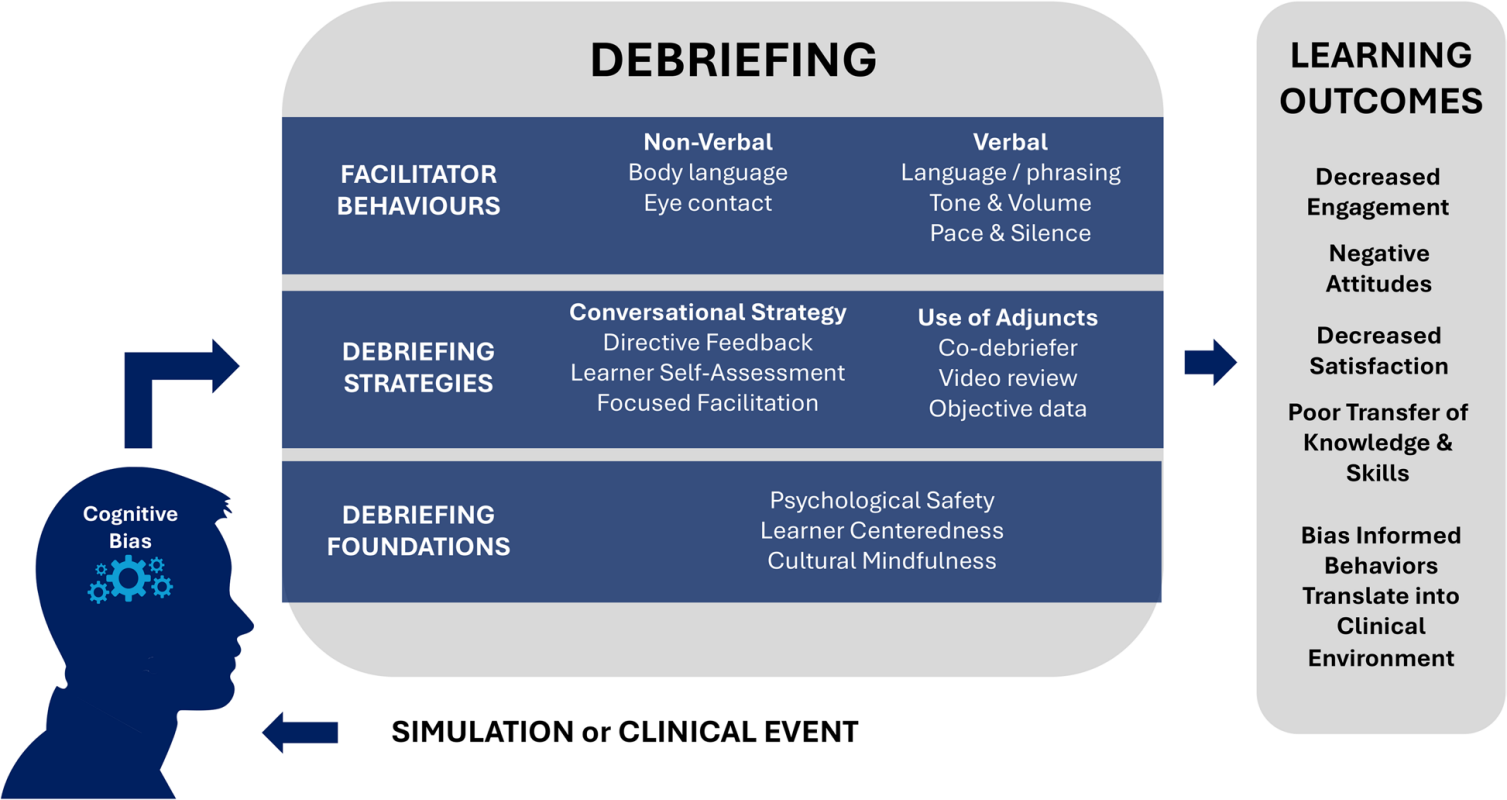
Impact of biases on performance and outcomes

Debriefing quality relates to how the debriefer engages the learners, the organization of the conversation, and the reflection it promotes [5, 11, 12]. Experienced debriefers are armed with a variety of tools including behaviors, conversational strategies as well as a focus on learner needs and safety. Despite our deliberate efforts, cognitive biases have the chance to unconsciously influence these approaches and ultimately risk learner outcomes as detailed in Fig. 1.

Fig. 1 Cognitive biases impact facilitator behaviors, debriefing strategies, and debriefing foundations, and when negative, risk learner outcomes in a variety of ways. The literature has outlined detailed behaviors like attentiveness to eye contact, tone, cadence, and the use of silence to mitigate difficult conversations and promote constructive dialogue. [13, 14]. A debriefer falling prey to cognitive biases may alter their paraverbal communication by increasing their cadence or tone, inadvertently silencing certain learners. Body language like leaning toward a learner can invite people into the debriefing [13]. Cognitive biases may produce the opposite effect including an unconscious favoritism toward one learner in a group expressed by the debriefer physically leaning in. Similarly, unconscious perceptions may cause the debriefer to lean away and avoid eye contact with other learners suggesting a lack of interest.

Greater reflection may be facilitated by the types of questions applied in the conversation or by adapting and modifying a strategy to promote learner needs and encourage problem-solving [3, 15–17]. Multiple constructs exist, including Debriefing With Good Judgment, Debriefing for Meaningful Learning, and the PEARLS Debriefing methodology among others, to intentionally create structure and flexibility so the facilitator has strategy options to best meet the needs of the learning situation [2, 18, 19]. Cognitive biases may create a perception mismatch of capabilities or knowledge of the learners resulting in a misdiagnosis of the situation and a prescribed learning approach that does not resonate or ostracizes the learners [20]. Debriefers’ unconscious assumptions can also impact how they emphasize certain aspects of the debriefing structure or highlight certain elements of a case through video or objective data while ignoring or avoiding others.

Underpinning reflective conversations is psychological safety; a shared belief held by members of a team that it is safe for interpersonal risk-taking (e.g., sharing perspectives, asking questions, speaking up with opposing opinions) without fear of retribution [21–23]. Maintaining psychological safety throughout the debriefing fosters a space for innovation, sharing ideas, advocacy, and willingness to participate, and contribute [22, 24]. Debriefing quality is also impacted by the competency of the debriefer, having both knowledge and skills associated with evidence-based debriefing practices [25] as well as cultural awareness of self and the learners with whom they are interacting. Lastly, a high-quality debriefing is anchored by the facilitator’s management of the balance between learner-centered and facilitator-centered teaching as they identify, explore, and close learning gaps to generate learning outcomes [20]. Cognitive biases can compromise these elements by creating an underappreciation of psychological safety breaches, a neglect of cultural differences in communication or an underestimation of learner needs.

Changes in debriefing behavior, strategies, or the loss of the foundational elements vital to debriefing conversations may ultimately impact learning outcomes. Learners may respond by decreasing engagement in the conversation, limiting the flow of constructive concepts. Negative attitudes and decreased satisfaction with the learning experience may build reluctance to return to simulation educational situations. Learners perceived negative experiences may also impair their ability to learn and leave learning objectives unmet [12]. Facilitator cognitive bias-influenced behaviors may translate beyond the learning environment into the clinical team. Relationally, biases targeting a learner as incompetent in debriefings may create distance between the learners who are part of the same clinical team inadvertently creating a challenging team dynamic that affects patient care. Clinically, biases that ignore certain underperformance due to favoritism of individuals or ideas, may create negative learning and influence decision-making at the bedside.

### Common biases in healthcare debriefing

There are hundreds of biases that have been identified that may impact debriefing conversations. It is not the purpose of this paper to classify them all but to approach awareness and discuss a sample of specific bias types that are closely linked to debriefing conversations that risk negative outcomes. It is also important to note that everyone has biases and that not all cognitive biases result in negative outcomes, as many of them are evolutionarily adaptive and help us navigate the world efficiently. The chosen biases selected are ones that the authors of this paper have identified over the last 15 years of experience as more common in debriefing conversations. The biases that follow include (1) fundamental attribution error, (2) halo/horn effect, (3) confirmation bias, (4) anchoring bias, (5) negativity bias, and (6) hindsight bias and are further delineated in Table 1.

**Table 1:**
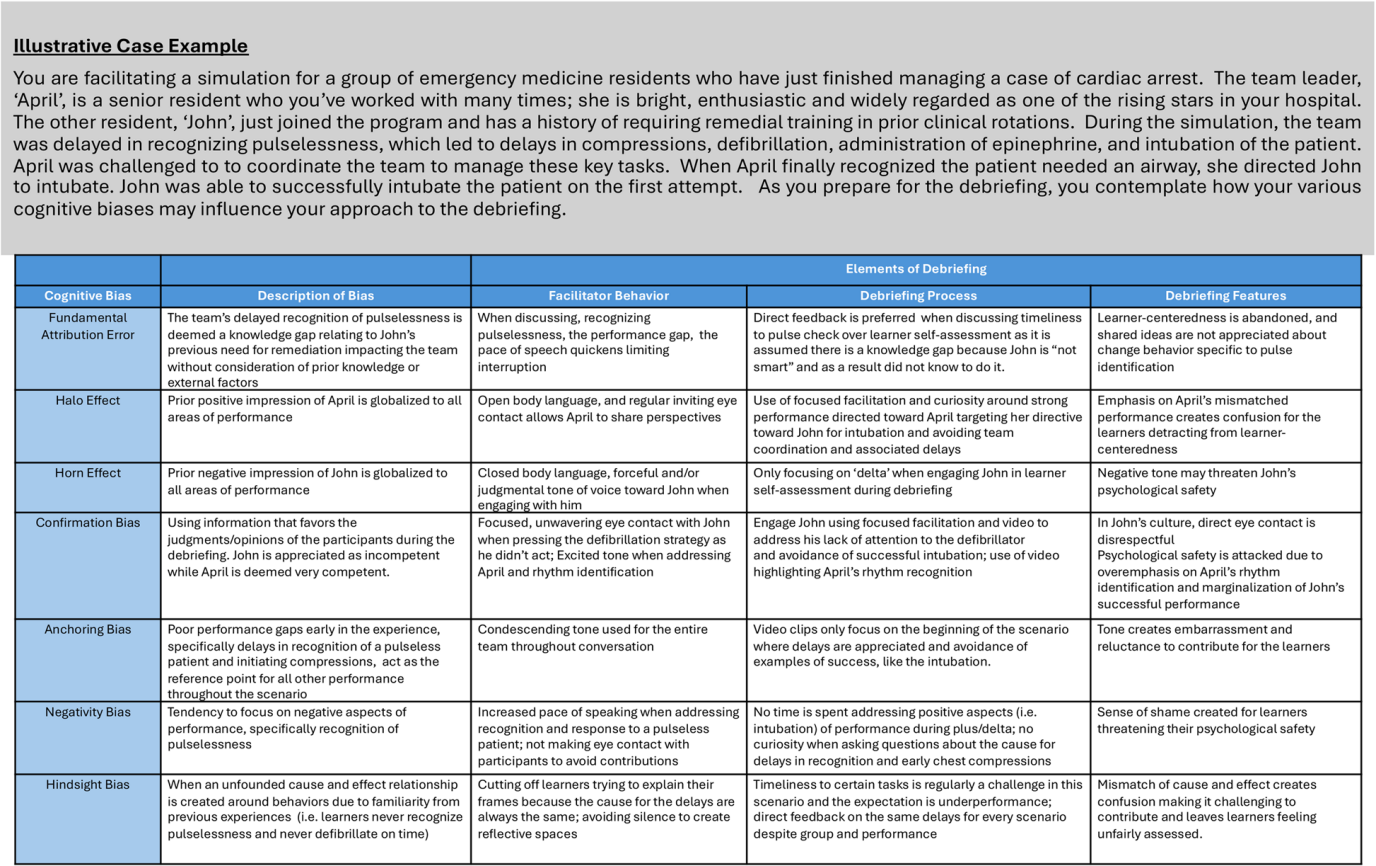
Illustrative case example of the impact of cognitive biases in debriefing conversations

### Fundamental attribution error

The fundamental attribution error is defined as when an individual overemphasizes personality-based factors, and downplays situational or environmental factors, in the evaluation and judgments about other people’s behaviors [26, 27]. In doing this, the observer may misinterpret the experience and inappropriately attribute a performance gap and appreciate it as a product of the individual’s personality, such as a learner’s previous need for educational support or remediation. More specifically in the context of debriefing, the fundamental attribution error may lead the debriefer to explain learner behavior based on internal attributes (personality factors or assumption of knowledge) rather than driver curiosity about the external attributes (context-based performance). During debriefing conversations influenced by the fundamental attribution error, debriefers interpret the actions they have seen during a scenario and enter one of two mental states. They can either be objective about the details of the event as they analyze, or lean into dispositional inference [26, 28], whereby the debriefer will not only evaluate the performance but integrate multiple inferences about the individuals’ values and characteristics into the evaluation.

Consider an example where a learner participating in a resuscitation did not begin chest compressions after a patient was found pulseless. The observer/debriefer recognized the behavior as underperformance, assuming the learner was inexperienced, and marked it as a topic for discussion. To target the perceived knowledge gap, a facilitator may increase their speech cadence and leave little room for response. The debriefer may alter their approach to asking questions during the analysis phase, choosing direct feedback over a focused facilitation technique that explores the frames of the learner [19]. In using direct feedback, the debriefer informs the learner of their knowledge gap of needing to immediately start compression per the American Heart Association Guidelines. Because of the fundamental attribution error, the debriefer has given misguided feedback as they interpreted the lack of compressions as being due to the trainee not knowing to start them expeditiously. By not seeking the frame of the learner or explore situational factors influencing performance with curiosity in this instance, the debriefer abandons learner-centeredness. The learner may in fact already be aware of this knowledge or have appreciated a pulse because the simulator malfunctioned. As a result, the debriefer may miss the growth opportunity to explore why the knowledge was not applied.

### Halo effect/horn effect

Although two separate cognitive biases, the halo and horn effect are best appreciated as a pair of opposites. The halo effect is defined as a cognitive bias that highlights a single positive attribute of an individual and unconsciously globalizes that attribute to all attributes of the individual, even when there is no evidence to support it [29]. In debriefing, the analysis of performance may be altered by the Halo Effect as it may inadvertently positively influence the evaluation of a learner in one area of knowledge or skill even though they have actually underperformed or have a lack of knowledge. Conversely, the horn effect is defined as a cognitive bias that impacts judgment about someone based on a perceived negative trait [30]. Like the halo effect, the horn effect unconsciously generalizes a negative attribute to all attributes of the individual thereby skewing global perception, potentially clouding judgment, and resulting in unfair assessments [31].

Halo effect can surface in numerous nuances in debriefing. For example, a healthcare team member or learner who shows enthusiasm or has a friendship with the debriefer may positively influence the debriefer’s judgment, even if this team member or learner lacks knowledge or competence in some areas [30, 32]. It also may impact the way a debriefer asks questions, potentially avoiding focused questions and curiosity about underperformance. Because of the halo effect, the process of evaluation is clouded by the previous positive relationship between the debriefer and the learner, leading the debriefer to categorize the learner’s poor performance as good behavior to avoid any potential rupture to the relationship.

As an example, a learner may perform high-quality chest compressions, and in another simulation struggle with defibrillator pad placement and team communication skills. Because of the halo effect, the debriefer may evaluate the learner’s competence across all skills based on the one positive attribute they have assigned to their high-quality chest compressions, neglecting to appreciate the poor performance in other areas of practice [33]. This may cause a focus on video review of only the chest compressions and an avoidance of the pad placement. This can ultimately lead to negative learning outcomes and confusion in learners who understand a practice standard was not met but the underperformance was instead commended or overlooked by the debriefer due to the influence of the halo effect bias.

The horn effect may similarly impact a debriefing, as a negatively associated learner attribute may change approaches to question-asking. From a lens of debriefing behavior, facilitators may choose language and phrasing that is less constructive. Normally a debriefer may convey curiosity through an effectively targeted advocacy inquiry to address a performance gap [19]. If the horn effect is influencing debriefing approaches, a debriefer may be more apt to avoid frame exploration and curiosity when advocating around a performance gap. This may leave a learner feeling frustrated as they are unable to surface the reasoning behind their performance, violating their sense of psychological safety, and potentially left unwilling to participate in future simulations or cloud their ability to focus and learn during the experience.

### Confirmation bias

Confirmation bias is defined as the unconscious favoring of information that strengthens the opinion or judgment of an individual. Under its influence, individuals seek out and assign greater value to their ideas, while avoiding and ultimately discounting or rejecting all other alternative explanations [34–36]. In a debriefing conversation, a facilitator unconsciously offers more weight or selective memory to supporting evidence during analysis that confirms their initial position or stance, rather than attending to other frames of reference.

Confirmation bias can show up in a debriefing in numerous ways. Once a debriefer has an idea or belief in a debriefing, the debriefer may intentionally seek out learners who will agree with their opinion. This may cause the debriefer to selectively make eye contact or call upon certain learners and ignore others. These beliefs may begin to influence debriefing strategies with an emphasis on how questions are formed. Confirmation bias may reveal itself by an inability to explore learners’ frames with true curiosity, or the asking of leading questions, or potentially derailment of learner discussion. In video-assisted debriefing, debriefers may choose specific performance moments of a scenario that support their vantage point and avoid using alternative moments that would be more beneficial for learners’ growth and development. In turn, learners may begin to perceive the debriefer as close-minded, which can breach psychological safety and shift the conversational dynamics. This bias could impact the accuracy of analysis, problem identification, and problem-solving during a debriefing and potentially lead to meeting fewer learning objectives and negatively impact the attitudes of the learners.

### Anchoring bias

Anchoring bias occurs when an individual relies on an initial impression or first piece of information and is unable to change their impression, even as new information becomes available [37, 38]. This initial piece of information becomes a reference point, or anchor, for making all other decisions or judgments. In other words, initial perception becomes the standard of comparison to judge or appraise all future perceptions and actions.

Because of anchoring bias, a debriefer may create an initial impression based on a learner’s or group’s early performance. If that early performance was a clinical misstep, an “anchor” may be created by the debriefer casting a negative impression. The team may excel through the rest of the experience; however, anchoring bias leaves the debriefer with an overall impression of underperformance. As a result, the debriefer may unconsciously avoid engaging, averting their eye contact with learners, as the anchor creates a lack of confidence in their knowledge base. Instead of strategizing to use a learner-centered approach like plus/delta, where positive behaviors can be applauded and reinforced the bias employs focused facilitation techniques without curiosity. Learners who are inappropriately targeted as a result of this bias can be confused by the mismatch of performance and negative feedback.

Alternatively, the “anchor” to their initial positive performance may bias the debriefer from fully observing subsequent missteps in the experience. This in turn may lead to missed opportunities to discuss performance gaps or lead the debriefer to subconsciously “forgive” or let slide other gaps. This also creates the potential for negative learning outcomes for learners as the unaddressed poor performance may be assumed to have met or surpassed a standard.

### Negativity bias

Negativity bias is defined as an unconscious favor to focus on negative experiences than positive ones within the learning environment [39]. Within simulation, emotional activation can serve as an enabler and a barrier to learning [40]. Simulation educators may need to determine if emotional activation is having a positive or negative impact on individual learners and the learning community. In educators and learners alike, negative emotional arousal, such as stress, can strongly influence performance as it requires the individual to divide attention, impairs working memory, and clouds decision-making [41]. These effects related to negativity bias, tend to more strongly influence our behavior than positive information [41]. Increased stress leads to greater negativity bias and impairs social skills [42], both of which may impact the facilitation of learning. Negativity bias has served an evolutionary benefit as it guides humans away from harmful situations [43]; within the context of simulation debriefing, however, it may bias the educators to stray from exploring and reinforcing positive behaviors in the environment and emphasize the negative ones in the learning experience [39].

To appreciate negativity bias in a debriefing, an educator may be influenced by a poor-performing team during a simulation, which colors their perception of the team during the debriefing. As a result, this bias may materialize in the form of body language as the debriefer takes on posturing or makes eye contact that suggests disapproval toward the learners. The educator inadvertently focuses attention during the debriefing on all the negative aspects of performance within the simulation as negativity dominance overpowers effective reflection and feedback [44–46]. Negativity dominance results in the educator neglecting the positive aspects of performance gains that may also be present, as the debriefer takes a direct feedback approach rather than a learner-centered approach [20] or a learning-from-success approach [47]. In doing so, the debriefer perpetuates their negativity bias, resulting in self-related negativity bias within their learners [47]. When negativity bias unconsciously influences the debriefer, it restricts the capacity for intellectual humility [48], this in turn may contribute to a learner not feeling psychologically safe to contribute to the debriefing conversation. This disrupted psychological safety has the potential to leave learners anxious about re-engaging in simulation-based learning and dejected about their capacity and contribution to work as a team in future cases [21].

### Hindsight bias

Hindsight bias occurs when an individual perceives an event as being more predictable after learning the correct or actual outcomes of an event [49]. Because of hindsight bias, an individual convinces themselves that they would have known the outcome of an event or solutions to a problem even if they had not been told the answers [49–51]. More simply, Fischhoff [52] referred to this phenomenon as the “knew-it-all-along” effect. In debriefing, the debriefer and learners may fall prey to hindsight bias and oversimplify the cause and effect of behavior after knowing the actual outcomes of a simulation scenario [53]. As a result, the debriefer may offer unfounded causes for an outcome and thereby limit reflection and potentially create confusion for learners during the conversation. This may impact the quality of the debriefing, as the debriefer is not identifying and targeting learning needs because variables contributing to certain actions or inaction may not be explored.

Debriefers are particularly vulnerable to hindsight bias in simulation as we tend to repeat scenarios where we often know the resolution or outcome of the case. The more frequently a debriefer is exposed to the same case over and over again the more difficult retrospective analysis becomes to recall their first experiences with the case [51]. Going into the debriefing, the debriefer may see the outcome of the scenario as known information, whereas learners experiencing it for the first time are more naïve to the outcome. Entering the conversation with outcome knowledge, the debriefer may judge the learners more harshly under the assumption that the learners should have known the correct course of action to take to arrive at the intended outcome. Influenced by hindsight bias, the debriefer may have a more difficult time appreciating the challenges and complexities the learners faced during the scenario, ultimately influencing debriefing strategies. A debriefer may approach the situation with focused facilitation with less curiosity or lean more toward instructor-centered teaching. This leaves the debriefing devoid of an analysis exploring actions taken or less consideration of other possible actions that may have been similarly appropriate. This recalling of an event based on previous experiences leads to discussing causal relationships that did not exist in the learning experience creating a mismatch and potential confusion for the learner [49] potentially influencing their learned management for clinical practice [53].

### Future directions

We have outlined how cognitive biases are a potential influence on every debriefing conversation and contribute to both positive and potentially negative experiences. By building awareness around our biases, we are beginning to attend to the homework debriefers need to better recognize and ultimately manage cognitive biases that have negative impacts. We recognize that we have only covered a small fraction of the hundreds of known cognitive biases and their multiple overlapping influences on debriefing conversations.

Both theoretical and empirical work is needed to further characterize and outline how awareness of these biases impacts debriefer performance, learner satisfaction, and learning outcomes, as well as potential ways to mitigate them. We hope this paper serves as a kick-starter for the self-exploration needed before debriefers can successfully venture into self-management during debriefing conversations and begin the exploration of strategy in the simulation community to build a culture for bias mitigation.

## Data Availability

No datasets were generated or analysed during the current study.
